# Development and Evaluation of Alginate Membranes with Curcumin-Loaded Nanoparticles for Potential Wound-Healing Applications

**DOI:** 10.3390/pharmaceutics11080389

**Published:** 2019-08-03

**Authors:** Mónica C. Guadarrama-Acevedo, Raisa A. Mendoza-Flores, María L. Del Prado-Audelo, Zaida Urbán-Morlán, David M. Giraldo-Gomez, Jonathan J. Magaña, Maykel González-Torres, Octavio D. Reyes-Hernández, Gabriela Figueroa-González, Isaac H. Caballero-Florán, Carla D. Florán-Hernández, Benjamín Florán, Hernán Cortés, Gerardo Leyva-Gómez

**Affiliations:** 1 Departamento de Farmacia, Facultad de Química, Universidad Nacional Autónoma de México, Ciudad Universitaria, Circuito Exterior S/N, Del. Coyoacán, Ciudad de México 04510, México; 2 Laboratorio de Posgrado en Tecnología Farmacéutica, FES-Cuautitlán, Universidad Nacional Autónoma de México, Cuautitlán Izcalli 54740, México; 3 Departamento de Biología Celular y Tisular, Facultad de Medicina, Universidad Nacional Autónoma de México (UNAM), Edificio “A” 3er piso, Circuito Interior, Avenida Universidad 3000, Ciudad Universitaria, Coyoacán, Ciudad de México 04510, México; 4 Laboratorio de Medicina Genómica, Departamento de Genética, Instituto Nacional de Rehabilitación Luis Guillermo Ibarra Ibarra, Ciudad de México 14389, México; 5 CONACyT-Laboratorio de Biotecnología, Instituto Nacional de Rehabilitación Luis Guillermo Ibarra Ibarra, Ciudad de México 14389, México; 6 Instituto Tecnológico y de Estudios Superiores de Monterrey, Campus Ciudad de México 14380, México; 7 Laboratorio de Biología Molecular del Cáncer, UMIEZ, Facultad de Estudios Superiores Zaragoza, Universidad Nacional Autónoma de México, Ciudad de México 09230, México; 8 CONACyT-Laboratorio de Genómica, Dirección de Investigación, Instituto Nacional de Cancerología. Av. San Fernando 22, Tlalpan, Sección XVI, Ciudad de México 14080, México; 9 Departamento de Fisiología, Biofísica & Neurociencias, Centro de Investigación y de Estudios Avanzados del Instituto Politécnico Nacional, Ciudad de México 07360, México

**Keywords:** wound dressing, polymeric membrane, nanoparticles, curcumin, alginate, pluronic F68, drug skin permeation, Franz cells, tape stripping

## Abstract

Non-biodegradable materials with a low swelling capacity and which are opaque and occlusive are the main problems associated with the clinical performance of some commercially available wound dressings. In this work, a novel biodegradable wound dressing was developed by means of alginate membrane and polycaprolactone nanoparticles loaded with curcumin for potential use in wound healing. Curcumin was employed as a model drug due to its important properties in wound healing, including antimicrobial, antifungal, and anti-inflammatory effects. To determine the potential use of wound dressing, in vitro, ex vivo, and in vivo studies were carried out. The novel membrane exhibited the diverse functional characteristics required to perform as a substitute for synthetic skin, such as a high capacity for swelling and adherence to the skin, evidence of pores to regulate the loss of transepidermal water, transparency for monitoring the wound, and drug-controlled release by the incorporation of nanoparticles. The incorporation of the nanocarriers aids the drug in permeating into different skin layers, solving the solubility problems of curcumin. The clinical application of this system would cover extensive areas of mixed first- and second-degree wounds, without the need for removal, thus decreasing the patient’s discomfort and the risk of altering the formation of the new epithelium.

## 1. Introduction

Human skin exerts a pivotal function as a protection barrier against diverse exogenous noxious factors; however, it is exposed and undergoes diverse types of injuries, including burns, ulcers, trauma, lacerations, and acute or chronic wounds, which may compromise its integrity [1].

In this regard, when skin is damaged, a specialized and highly regulated dynamic process immediately takes place: wound healing [2]. The main goal of wound healing is to restore tissue integrity and to achieve homeostasis; however, this process may be complicated by distinct intrinsic and extrinsic factors [2]. Thus, in order to accelerate wound healing, a variety of wound dressings have been designed. 

Irrespective of the type of wound, the main function of dressings is to aid in the repair of the wound through the reduction of pain and inflammation, to protect the damaged tissue from pathogenic agents, and to enhance cell differentiation and proliferation [3]. Although there several types of dressings which are commercially available, many of these present some drawbacks, such as an inefficient absorption of exudates, poor protection against infections by microbes, the lack of ability to maintain humidity, and the triggering of allergic effects [4]. In addition, several dressings may adhere to the wound and require constant changes, which may interfere with the granulation process and delay the healing course.

Therefore, in recent years, there has been increasing interest in asymmetric membranes as an alternative for designing wound dressings [5]. These types of membranes possess multiple advantages, such as structural similarity with the skin, an ability to absorb exudates due to their porous structure, and improved cell adhesion and proliferation [6,7]. Different polymers have been employed for their development, including chitosan, hyaluronic acid, collagen, poly vinyl alcohol (PVA), polycaprolactone (PCL), and alginate [8]. In particular, alginate is a biopolymer extracted from seaweed and has shown several unique properties, such as biodegradability, good hydrophilicity, and good biocompatibility [4]. Alginate has exhibited potential for improving wound healing due to its hemostatic properties; moreover, it may reduce microbial infections, enhance the absorption of exudates, and decrease allergic reactions [9]. These features render alginate an interesting option for wound dressings. In addition, alginate membranes possess the advantage that they may be functionalized with bioactive compounds that enhance their healing properties.

In this regard, curcumin is a natural compound that possesses a plethora of biological activities, including antimicrobial, antifungal, anti-inflammatory, and antioxidant effects [10,11,12]. In addition, curcumin improves wound healing, enhances epithelial regeneration, and increases the proliferation of fibroblasts [13,14]. Thus, curcumin could be a suitable pharmacological agent for the elaboration of wound dressings. However, curcumin exhibits low bioavailability and is unstable in neutral and alkaline aqueous solutions, as well as in hydrophilic topical preparations [12,15]. These drawbacks could be overcome by a nanoparticle formulation that permits the controlled and gradual release of the compound. 

Therefore, the objective of this study was to design and develop a novel wound dressing comprising an alginate membrane and PCL nanoparticles loaded with curcumin and stabilized with Pluronic^®^ F-68 (CNp) for possible application in wound healing. The wound dressing was physicochemically characterized, and in vivo and ex vivo permeation assays were performed.

## 2. Materials and Methods 

### 2.1. Materials 

For CNp fabrication, we used PCL (Mn 80,000 g/mol), Pluronic^®^ F-68, and trehalose dihydrate, which were purchased from Sigma-Aldrich^®^ (Merck KGaA, Darmstadt, Germany), whereas methanol, ethyl acetate, and curcumin were supplied by Spectrum^®^ (Spectrum Laboratory Products, CA, USA). For membrane elaboration and characterization, we employed sodium alginate [(SA) (300–700 cps 1.0%, RV, 20 rpm, 25 °C], glycerol (Gly), propylene glycol (Prop), and Tween 80, which were purchased from Droguería Cosmopolita (Mexico City, Mexico). PVA, Pluronic^®^ F-127, acetone, and phosphate buffered saline (PBS) solution were supplied by Sigma-Aldrich^®^ (Merck KGaA, Darmstadt, Germany). Kollidon^®^ 30 [Polyvinylpyrrolidone k 30; (PVP k30)] was obtained from BASF (USA). Ethyl acetate was acquired from Distribuidora Química Alvi (State of Mexico, Mexico). Methanol was purchased from Fermont (Nuevo León, Mexico). All other chemicals and reagents were of at least analytical-grade quality.

### 2.2. Preparation of PCL Nanoparticles Loaded with Curcumin (CNp)

CNp were prepared by the emulsification–diffusion method, as previously described by Quintanar-Guerrero, et al. [16]. Briefly, the organic saturated phase and aqueous saturated phase were obtained by the saturation of an ethyl acetate and distilled water mixture at a 1:1 ratio. After that, 2% (*w*/*v*) solution of PCL was prepared by dissolving 400 mg of PCL in 20 mL of the organic saturated phase; once PCL was dissolved, 100 mg of curcumin was added. At the same time, 5% (*w*/*v*) of Pluronic^®^ F-68 solution was prepared using an aqueous saturated phase as solvent. In order to obtain an emulsion, both solutions were mixed at a 1:2 ratio with a high-speed homogenizer (Ultra Turrax T18; IKA^®^) at 14,000 rpm for 10 min at room temperature. Then, 160 mL of water was added to the emulsion to generate polymer aggregation, and the system was maintained under the same conditions for 10 min. The organic solvent was evaporated by a rotary vacuum (Heidolph^®^, Schwabach, Germany), and the nanoparticle suspension obtained was centrifuged at 15,557 *g* for 30 min at 25 °C. Finally, the pellet was dissolved in distilled water. In order to assess the thermal stability and the properties of the nanoparticles, CNp were frozen and lyophilized at −49 °C, 0.05 mBar for 24 h, employing 5% *w*/*v* trehalose dihydrate as a cryoprotectant.

### 2.3. Physicochemical Characterization of CNp

#### 2.3.1. Particle Size and Zeta Potential Assessment

The particle size and distribution (polydispersity index, PDI) of the CNp were evaluated by dynamic light scattering. On the other hand, to determine the Zeta potential of CNp, laser Doppler velocimetry was employed. CNp dispersions were assessed five times at 25 °C in a Zetasizer (Malvern Instrument ZS90; Malvern, UK).

#### 2.3.2. Atomic Force Microscopy (AFM) 

CNp size and geometry were analyzed by atomic force microscopy (AFM) with a scanning probe microscope (JSPM-4210, JEOL^®^, Tokyo, Japan). In brief, the CNp dispersion was obtained after centrifugation, and the pellet resuspension was diluted to 1:100 with distilled water. A drop was placed on a coverslip, allowing it to dry at room temperature. The coverslip with the drop was held in place with carbon tape, and room-temperature conditions were utilized to assess the samples. 

#### 2.3.3. Drug Loading and Entrapment Efficiency of CNp

To calculate entrapment efficiency (EE) and drug loading (DL), the CNp dispersion was centrifuged at 15,557 *g* for 40 min; then, the sediment was resuspended in ethyl acetate and the absorbance was measured by UV–Vis spectrophotometry at 420 nm (DLAB^®^, SP-UV1000, Beijing, China). The amount of curcumin was obtained by interpolation in a calibration curve (*R*^2^ coefficient = 0.99985). 

The percentages of EE and DL were calculated from the equations below:

(1) $$\%\;\mathrm{EE}=\;\frac{\mathrm{CN}}{\mathrm{IC}}\times 100$$

(2) $$\%\;\mathrm{DL}=\;\frac{\mathrm{CN}}{N}\times 100$$

where CN = the amount of curcumin in nanoparticles, IC = the initial amount of curcumin, and N = the number of nanoparticles.

### 2.4. Preparation of Polymer Gels and Membranes

Four membrane formulations based on SA (M1, M2, M3, and M4) (See Table 1) were prepared using the solvent casting method as published by Karki et al. [17]. First, SA and the polymer were dissolved separately in injectable water by stirring at 35 °C. After dissolution, they were mixed with each other by mechanical stirring. Then, the plasticizer (or a plasticizer mixture) was added by stirring at room temperature until a homogeneous gel was obtained. In order to eliminate bubbles from the gel, it was centrifuged at 636 *g* for 20 min at room temperature (BIOBASE, BKC-TH18II, Shandong, China). 

In order to obtain the membranes, 10 g of each gel, prepared with the previously mentioned methodology, was poured into a Teflon cast 12 cm in diameter and left to dry into an oven (OAKTON Stable Temp, IL, USA) at 75.0 ± 0.5 °C for 3 h.

#### 2.4.1. Preparation of Nanoparticle-Coated Alginate Membranes (CNp‒M4)

The methodology described previously was followed to prepare our nanoparticle-coated alginate membrane (CNp‒M4), but the vehicle utilized was a dispersion of CNp to obtain 0.01% *w*/*v* of curcumin instead of injectable water. In order to prepare the membranes, the gel obtained was poured into a Teflon cast and left to dry in an oven at 40.0 ± 0.5 °C for 4 h. 

### 2.5. Physicochemical Characterization of Membranes

#### 2.5.1. Swelling Test

Samples of M1, M2, M3, M4, and CNp‒M4 membranes were cut to a size of 1 × 1 cm and weighed on pre-weighed aluminum trays. The samples were divided into five blocks, corresponding to different times as follows: 5, 10, 20, 30, and 60 min. The membranes on the trays were placed on a flat surface, and 750 μL of PBS 1X pH 7.4 was added to each sample. 

Once the established time had elapsed, the trays were turned vertically for 2 min on absorbent paper, allowing the draining and absorbption of the excess of PBS. After that time, the membranes in the trays were weighed again [18]. The assessment was performed in triplicate for each of the different times. 

The swelling percentage (%S) was calculated using the following formula [19]:

(3) $$\%S=\;\frac{M_{s}-M_{d}}{M_{d}}\times 100$$

where $M_{s}$ and $M_{d}$ are the weight of the swollen membrane and dried membrane, respectively.

#### 2.5.2. Mechanical Test

Tensile strength (TS) and the elongation at a break (%E) of M1, M2, M3, M4, and CNp‒M4 were determined using a Sintech ½ testing machine (MTS, USA), which was equipped with a 100-N load cell at a crosshead speed of 2.4 mm s^−1^. Three samples of each formulation were cut into a dumbbell shape with a width of 10 mm and an effective length of 40 mm between the clamps at the beginning of the measurement. The thickness of each sample was measured using a Vernier at five different points before testing, and the average of these was employed for TS calculation. The load (Lb) and displacement (mm) of each film were recorded during the stretching. TS and %E were calculated by Equations (4) and (5), as published by Karki et al. [17]:

(4) $$\mathrm{TS}\;=\;\frac{F}{A}$$

(5) $$\%E=\;\frac{D}{L}\times 100$$

where TS is reported in MPa, F is the maximum load (N) required to break the film, and A is the initial cross-sectional area in mm^2^. In Equation (5), D is the displacement of the film elongation at the rupture and L is the initial length. 

#### 2.5.3. Thermogravimetric Analysis (TGA)

The thermal stability of the membranes (M4, CNp‒M4), CNp, and curcumin was evaluated through TGA, employing a Hi-Res TGA 2950 Thermogravimetric Analyzer (Modulated TA Instruments, New Castle, DE, USA). In brief, 5 mg of each sample was analyzed starting at room temperature and increasing to 500 °C at a heating rate of 10 °C/min under a nitrogen atmosphere. 

#### 2.5.4. Differential Scanning Calorimetry (DSC) 

The thermal properties of the membranes (M4, CNp‒M4), CNp, and curcumin were determined with the DSC 2910 (Modulated TA Instruments, DE, USA). Lyophilized samples were placed in hermetic aluminum cells and evaluated starting at room temperature and increasing to 250 °C at a heating rate of 10 °C/min under a nitrogen atmosphere. 

#### 2.5.5. pH Values

The pH values of M1, M2, M3, M4, and CNp‒M4 polymer gels was measured through a pH meter. The samples were put into contact with the electrode until a constant value was obtained. The pH meter was previously calibrated against standard solutions to ensure the highest level of accuracy. 

#### 2.5.6. Structure and Morphology of M4 and CNp‒M4 Membranes

In order to analyze the structure and morphology of the membranes, samples were cut in a circular shape 1 cm in diameter and in a longitudinal section and were analyzed using a scanning electron microscope (Jeol-JCM 6000, MA, USA) at 100× and 220× magnification; then, photographs were taken in four different fields. 

The pore diameter and membrane width were measured using a software package (ImageJ, MD, USA), and the average was calculated. Pore number was counted field-by-field, and the average was determined. 

In order to evaluate the transparency of M4 and CNp‒M4 membranes, samples were cut into a circular shape 3 cm in diameter and these were placed over an image (before swelling); then, they were observed and photographed. Afterward, 1 mL of PBS 1X was added to the samples, and after 20 min (swelling process), photographs were taken of the samples. 

#### 2.5.7. In Vitro Release Study of Drug Dispersion, CNp and CNp‒M4 Membrane

To evaluate the curcumin release profile from the CNp and CNp‒M4 membrane, the direct dispersion method was applied. For the drug and CNp dispersion, a certain amount of curcumin (and the equivalent in nanoparticles) was dissolved into PBS solution (pH 7.4, Pluronic^®^ F-127 2% *w*/*v*), divided into sets of three tubes each, and placed in a shaker incubator, maintaining this at 37 °C. For the CNp‒M4 membrane, disks 1 cm in diameter were placed in tubes with the PBS solution under the conditions previously described. At the defined times of 0, 0.25, 0.5, 1, 2, 4, 6, 24, and 48 h, one set of tubes was removed from shaking and centrifuged at 18,514 *g* for 30 min. The curcumin released in the supernatant was quantified by UV-Vis spectrophotometry at 420 nm.

The results of the release tests of CNp and CNp‒M4 membrane were analyzed by mathematical models such as zero-order, first-order, the Higuchi model, and the Korsmeyer–Peppas model to predict the drug release mechanism.

### 2.6. Permeation Assays

#### 2.6.1. Ex Vivo Permeation Assay

Porcine skin was obtained from the back of pig ears within 12 h after slaughter. The pig ears were cut into circular sections 3 cm in diameter. The excess of fat was removed with surgical scissors, and the samples were washed with saline solution. The samples were divided into three groups: drug dispersion with 0.01% *w*/*v* of curcumin (2 mL; *n* = 3), CNp dispersion with 0.01% *w*/*v* of curcumin (2 mL; *n* = 3), and CNp‒M4 membranes (3 cm in diameter; *n* = 3).

The experiments were conducted in 12 independent Franz cells with a diffusion area of 7.07 cm^2^. The freshly excised skin was placed into Franz cells, and the stratum corneum remained in contact with the donor compartment, with the dermis facing the receptor compartment. This was filled with 30 mL of 0.1 M PBS solution (pH 7.4) with 2.5% of Tween 80 and maintained under constant stirring at 400 rpm.

Franz cells were immersed in a water bath at a constant temperature of 37.0 ± 0.5 °C. At predetermined times (1, 2, 3, 4, 5, 6, 7, 8, 22, 24, 26, 28, and 30 h), 1000 μL of medium was removed from the receptor compartment and replaced with 1000 μL of fresh receptor medium.

At the end of the test, the skin samples were carefully removed from the Franz cells to conduct the tape-stripping test, following the in vivo permeation assay methodology (described in Section 2.6.2). All tapes were placed in a flask with 40 mL of acetone and mechanically stirred for 15 h. Subsequently, each skin sample was fragmented into small pieces using surgical scissors. The curcumin was extracted with 25 mL of ethyl acetate:methanol at a ratio of 9:1. The extract was centrifuged at 10,174 *g* for 10 min. All samples were analyzed by UV-visible spectrophotometry at a wavelength of 420 nm. 

#### 2.6.2. In Vivo Permeation Assay

To evaluate the in vivo permeation of the CNp‒M4 membrane through the stratum corneum, the tape-stripping technique was employed. Four healthy Mexican males aged 22–36 years were recruited as volunteers. The individuals had neither a history of skin disorders nor had used cosmetic products on their forearms 24 h prior to the test. Written informed consent was obtained from each volunteer before each study. 

Four sites (3 × 3 cm) were demarcated: two on the right forearm and two on the left forearm. Before administering the treatment, the sites were cleaned with cotton impregnated with water. Three treatments were administered to each volunteer. The CNp dispersion with 0.01% of curcumin (2 mL) and the drug dispersion at the same concentration were contained within a glass cylinder on the left forearm. The CNp‒M4 membrane (3 cm in diameter) was put on the right forearm, and 1.75 mL of distilled water was added onto the surface of the membrane. The last site on the right forearm was used for the blank. The treatments were in contact with the skin for 6 h [20]. Once the time had elapsed, the membrane was removed with steel nippers. The skin was subjected to 15 successive tape strips (Scotch^®^ 3M^®^), previously cut into 3 × 3 cm squares. In each case, the site was pressed uniformly by sliding a spatula over the surface of the tape five times; then, it was removed by pulling it with steel nippers from the lower right to the upper left end. The blank site was subjected to the same procedure. Each tape was immersed in 15 mL of acetone in a different amber glass bottle with a lid. All of the bottles were placed under mechanical agitation for 15 h; subsequently, the samples were filtered to remove the glue particles. The quantification of curcumin was performed by UV–visible spectrophotometry at a wavelength of 420 nm. Each sample was analyzed in triplicate. 

## 3. Results and Discussion

### 3.1. Physicochemical Characterization of CNp

#### 3.1.1. Particle Size and Zeta Potential Assessment

CNps were obtained, and their mean particle size and PDI were 148.3 ± 1.9 nm and 0.044 ± 0.020, respectively. These values were expected to improve dermal permeation [21], since nanoparticles below 500 nm have a larger surface area-to-volume ratio, which ensures direct contact with the stratum corneum and skin appendages [6,7]. Moreover, the PDI value was below 0.1, which indicates that the small size measured in the sample is reliable and monodisperse [22].

On the other hand, CNp exhibited a zeta potential value of −7.32 ± 0.03 mV. In this regard, zeta potential is commonly employed to measure the charge in the nanoparticles and/or electrostatic repulsion [1], and the literature indicates that nanoparticles are stable in suspension with a zeta potential above ±30 mV [23]. Despite the CNp zeta potential value not being in this range, it should be considered that Pluronic^®^ F-68 was added as a stabilizer of nanoparticles, which provides them with stability by means of a repulsion effect through a steric mechanism [24]. Likewise, CNp possesses a negative charge, which is related to the carboxylic end group of PCL [25]; thus, negatively charged nanoparticles could permeate adequately in conjunction with the negative charges existing on the skin [26].

#### 3.1.2. Atomic Force Microscopy (AFM)

**Figure 1:**
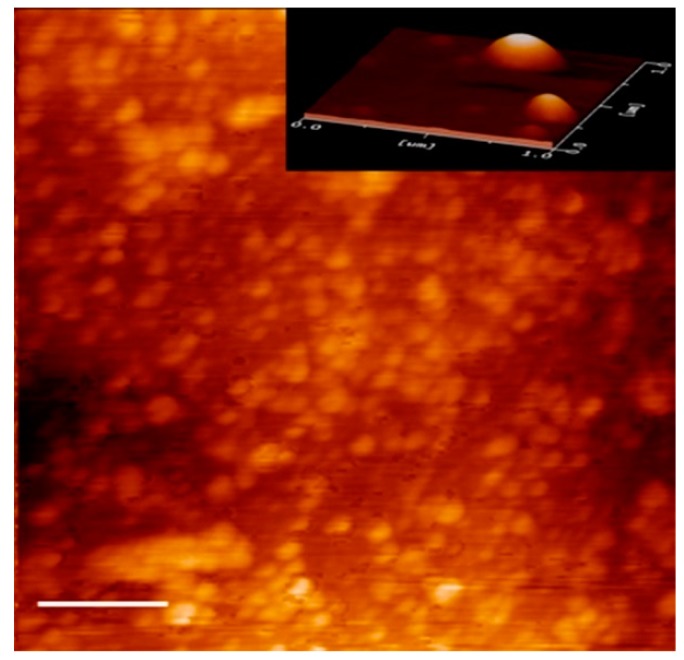
Atomic force microscopy (AFM) topography images of the CNp. The size bar is 1 µm.

The morphology and size of CNp were evaluated by AFM. In agreement with the particle-size and zeta-potential assessments, the nanoparticles demonstrated a spherical shape and a size of approximately 200 nm with no agglomeration (Figure 1) [27]. These results support the idea that the small size of CNp could improve the dermal permeation of curcumin, which would increase its anti-inflammatory, antimicrobial, and wound-healing activities [6,12].

#### 3.1.3. Drug Loading and Entrapment Efficiency of Nanoparticles 

In order to determine the amount of curcumin entrapped inside the CNp, the DL and EE were determined. The CNp showed DL and EE values of 4.9 ± 0.7% and 96.01 ± 0.95%, respectively. It is known that the emulsification diffusion method ensures high encapsulation efficiencies (generally >70%) [28]. On the other hand, DL depends on nanoparticle structure and methodology [29], whereas both DL and EE depend on the interactions between the drug, the matrix, and the medium [29]. 

### 3.2. Physicochemical and Mechanical Characterization of Nanoparticle-Coated Alginate Membranes as Wound Dressings

#### 3.2.1. Swelling Test

**Figure 2:**
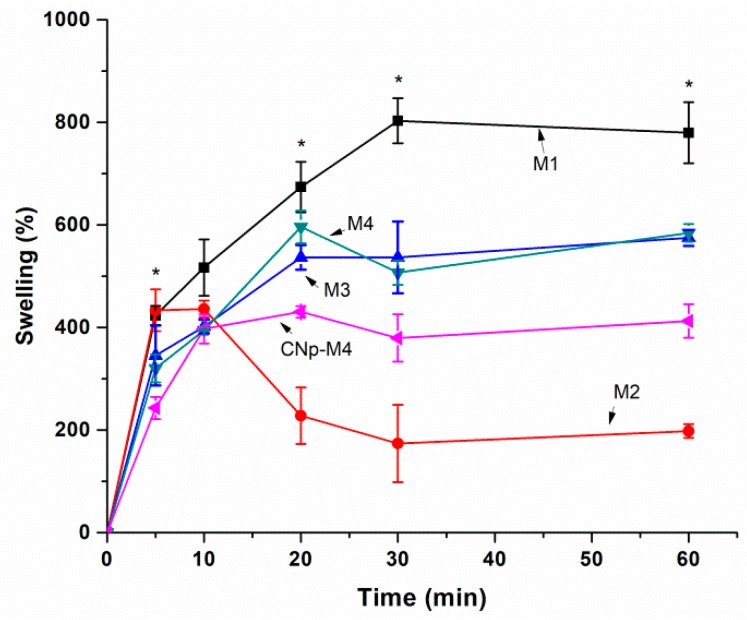
Percentage of swelling in alginate membranes as a function of time. Effect on swelling capacity by the addition of PVA + glycerin (**M1**), PVA + glycerin + propylene glycol (**M2**), PVP + glycerin (**M3**), PVP + glycerin + propylene glycol (**M4**), and CNp + PVP + glycerin + propylene glycol (**CNp‒M4**) (mean ± SE; *n* = 3). There were significant differences when ANOVA was applied at 5, 20, 30, and 60 min (*p* < 0.05).

From a practical point of view, the membranes should absorb the exudate from the wound and at the same time provide a moist environment that promotes healing. For this reason, the percentage of swelling of five different formulations was determined by weight difference (Figure 2).

Figure 2 depicts a difference at a time of 20 min, when propylene glycol is added to the formulation with PVA (M2) in comparison to M1; however, there is no significant difference between using or not using propylene glycol in the formulation combined with PVP (M3 and M4, respectively). In order to determine the differences in the percentage of swelling according to each formulation as a function of time, an ANOVA was performed. It was demonstrated that both the formulation and the time during which the membrane was exposed to PBS exerted a statistically significant effect on swelling with a 95% confidence level.

The formulation with the highest swelling value was M1, at 30 min (802.8 ± 76.0%), while the addition of propylene glycol (M2) decreased the swelling percentage after 10 min. On the other hand, M2 showed the lowest value in the swelling test before dissolving. The addition of propylene glycol to the polyvinyl alcohol mixture could increase the intermolecular interactions between both excipients via hydrogen bonds in the –OH groups, favored by a wide steric disposition, but could decrease the entry capacity of water molecules and their interaction with alginate by saturation, decreasing the swelling ability. In the case of the interaction of propylene glycol and PVP, the situation could be the opposite. The interaction of both excipients is lower, resulting in a lower swelling capacity of the alginate; i.e., the excipient that determines the majority of the response. This phenomenon is confirmed with the profile observed for CNp‒M4 in relation to M4. 

Namely, when water enters the polymer matrix, the chains begin to relax, giving rise to the opening of the polymer networks. This promotes the penetration of more water; however, in the last stages of swelling, the diffusion coefficient is diminished because the chains are completely relaxed and near equilibrium [30].

The membranes began to dissolve after being exposed to PBS for a longer period of time. According to the composition of the medium, polymers undergo degradation and erosion processes. When a polymer degrades, the chains are cleaved into oligomers and subsequently monomers. The continuous loss of monomers will eventually lead to the phenomenon of erosion, which progressively changes the microstructure of the membrane through the formation of pores [31]. The combination of these processes could favor the possible application of our M4 and CNp‒M4 membranes as wound dressings, because it would not be necessary to remove them from the application site, avoiding harm through injury and discomfort to the patient.

Moreover, the level of exudate from a wound (for example, an ulcer) can vary from absent (dry ulcer) to minimally exuding (<5 mL fluid per 24 h), to moderately exuding (5 to 10 mL fluid per 24 h), and finally to highly exuding (>10 mL fluid per 24 h) [32]. In this regard, the measurement of the swelling capacity of a wound dressing developed with alginate could be classified as dressings of low absorbance (alginate wound dressing that absorbs less than 6 g of liquid per g of dressing, or less than 12 g/100 cm^2^), and dressings of high absorbency (an alginate wound dressing that absorbs 6 g or more liquid per g of dressing, or 12 g or more/100 cm^2^) [33]. With this consideration, CNp‒M4 possesses a value of 17.48 g/100 cm^2^, corresponding to high absorbency and similar to several commercial products.

#### 3.2.2. Mechanical Test

**Figure 3:**
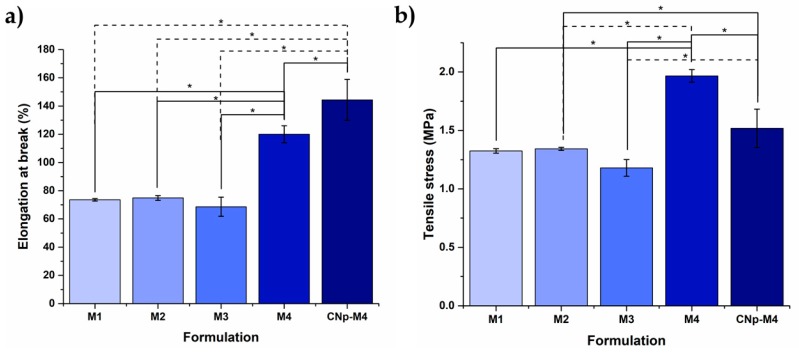
Effect of plasticizers on the mechanical properties of alginate membranes. Effect on mechanical properties by the addition of PVA + glycerin (M1), PVA + glycerin + propylene glycol (M2), PVP + glycerin (M3), PVP + glycerin + propylene glycol (M4), and CNp + PVP + glycerin + propylene glycol (CNp‒M4), respectively. (**a**) Elongation at break; (**b**) tensile stress, (mean ± SD; *n* = 3). * indicates *p* < 0.05 as statistically significant.

Wound dressings must be resistant and flexible for ease of handling [17]. Thus, the mechanical properties of the membranes are depicted in Figure 3. Formulations with PVA (M1 and M2) did not demonstrate a significant difference in %E with 73.54 ± 0.87% and 74.90 ± 1.67%, respectively. TS was similar for M1, M2, and M3 samples with 1.32 ± 0.02, 1.34 ± 0.01, and 1.96 ± 0.05 MPa, respectively. By way of comparison, M1 and M2 membranes exhibited a lower TS and %E than the remaining formulations, probably due to PVA being a polymer that has been characterized as possessing poor elasticity, a rigid membrane, and low hydrophilic characteristics [34]. 

The %E and TS of M3 were 68.63 ± 6.75% and 1.18 ± 0.07 MPa, respectively; these values significantly increased with the addition of propylene glycol (M4), to 120.01 ± 5.97% and 1.96 ± 0.05 MPa, respectively. M4 exhibited the highest values in the assay and showed a significant difference with respect to the remaining formulations without CNp. This may be explained, at least in part, by the properties of propylene glycol, which is a plasticizer with a small molecular weight that is able to create multiple H-bonds with PVP and SA chains into a package and, consequently, aid in the formation of cross-linked networks [35]. 

Therefore, because of its greater swelling capacity and better mechanical properties, the M4 membrane (a mixture of sodium alginate, PVP, and propylene glycol) was chosen for the incorporation of CNp.

On the other hand, the mechanical properties of M4 were modified when CNp dispersion was added to the formulation. The %E of CNp‒M4 showed the highest value, with 144.39 ± 14.52%; in contrast, TS decreased to 1.52 ± 0.16 MPa. This could be due to the addition of CNp dispersion to the formulation decreasing the number of hydrogen bonds between the polymer molecular chains; as a result, less strength is necessary to break the membrane [36]. In comparison, CNp‒M4 showed the highest %E, which could be due to the effect of Pluronic^®^ F-68. The latter is a surfactant that decreases the pore number, providing a membrane with a homogeneous structure; thus, it is more resistant to changes, rendering higher elasticity properties.

#### 3.2.3. Thermogravimetric Analysis (TGA)

**Figure 4:**
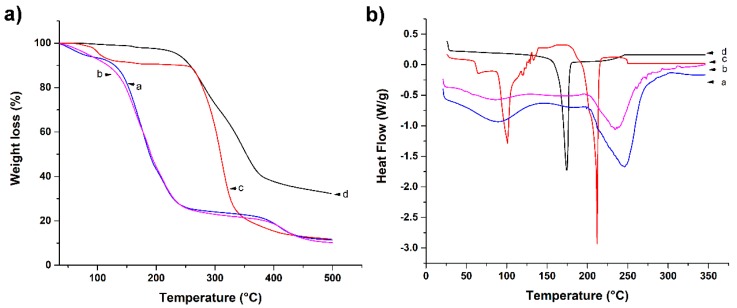
Thermal analysis of nanoparticle-coated alginate membrane using (**a**) thermogravimetric analysis (TGA) and (**b**) differential scanning calorimetry (DSC). The thermal properties of the M4 membrane, CNp‒M4 membrane, CNp, and curcumin are represented as a, b, c, and d, respectively.

It is important to determine the thermal properties of a substance, because these provide useful information for their identification and the characterization of materials. In Figure 4, thermograms of curcumin, CNp, M4, and CNp‒M4 membranes are presented. For curcumin, mass loss was observed at 173 °C by TGA (Figure 4a); due to the degradation of turmeric powder, water loss was not observed, possibly due to its high hydrophobicity [37]. The weight loss of CNp started at approximately 90 °C, corresponding to dehydration, and there was a second plateau from 280 to 350 °C, suggesting a better thermal stability for curcumin when it is inside PCL nanoparticles than when alone. However, CNp lost more weight in a smaller temperature range.

On the other hand, in the M4 membrane, the first mass loss occurred between 90 and 240 °C, whereas for the CNp‒M4 sample, weight loss began between 100 and 240 °C. This could be due to the evaporation of water traces, the degradation of propylene glycol, PVP, glycerin (150‒220 °C), and turmeric powder [38]. In the case of the CNp‒M4 membrane, it presented a slighter weight loss compared to the M4 membrane. The second mass loss of both samples was between 270 and 425 °C; in this stage, the decomposition of the functional groups of SA polymer chains is presented. Finally, the last plateau in M4 and CNp‒M4 membranes started at 425 °C, which corresponds to the degradation of the SA backbone [19]. In the same manner, the CNp‒M4 membrane thermogram revealed a lower weight-loss temperature compared with that of CNp; this is probably because membrane formulation is a mixture that contains more substances than CNp. This is similar to the thermal behavior exhibited by curcumin and CNp. 

#### 3.2.4. Differential Scanning Calorimetry (DSC)

DSC is a technique used to determine the quantity of heat either absorbed or released when substances undergo physical or chemical changes [39]. In Figure 4b, DSC thermograms of curcumin, CNp, M4 membrane as vehicle, and CNp‒M4 membrane are presented. The melting point of curcumin was found to be 174 °C (Table 2), which was expected with regard to the literature [40]. Furthermore, three thermal events were observed in the CNp: at 63.5; 101, and 212 °C. The first thermal event could correspond to the melting point of PCL (61 °C) [41], while events at 101 and 212 °C may indicate the presence of trehalose, which was employed as a cryoprotectant to lyophilize the CNp [42]. Interestingly, the melting point of curcumin was not detected in the CNp sample; this could be due to the high EE of curcumin inside PCL nanoparticles as a molecular dispersion. 

On the other hand, both M4 and CNp‒M4 membranes revealed two thermal events. The first peak was around 87 °C for both formulations, whereas the second peak was around 233 °C for the M4 membrane and 249 °C for the CNp‒M4 membrane. The latter peaks were due to the presence of SA in the formulation. A thermal peak prior to 100 °C was observed for both samples, possibly due to the presence of water in the membranes. 

#### 3.2.5. pH Determination

The pH values of all the polymer gels were 5.78 ± 0.06, 5.76 ± 0.03, 5.65 ± 0.01, 5.97 ± 0.05 and 5.68 ± 0.03 for M1, M2, M3, M4, and CNp‒M4, respectively. All of these values are acceptable because pH wound dressings must be neither acid nor alkaline in order to avoid skin irritation. Moreover, membrane pH is important to regulate the wound-healing process. The natural pH of the skin is within a range of 5–6, depending on the person, while the pH of the chronic wound oscillates in an alkaline range between pH 7 and 8, which increases susceptibility to wound infection. 

#### 3.2.6. Structure and Morphology of M4 and CNp‒M4 Membranes

**Figure 5:**
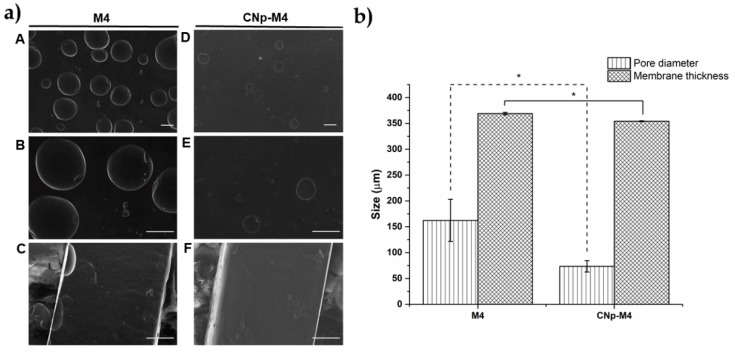
Morphology and porosity of alginate membranes. (**a**) Micrographs by scanning electronic microscopy of the alginate membrane surface (A, B, D, and E) and membrane thickness (C and F). Magnification of 100× for A, D; 220× in B, C, E and F; the scale bar is 100 μm; (**b**) pore diameter and membrane thickness of M4 and CNp‒M4 membranes, mean ± SE, *n* = 3. * indicates that *p* < 0.05 is statistically significant.

An ideal scaffold is expected to have a suitable microstructure (number of pores and pore size controlled) in order to transport nutrients, cells, metabolites, gases, and signaling molecules [43]. In this respect, pores were observed in the top of the membrane structure, which did not span the membrane (Figure 5a). However, it should be expected that the addition of water to the membranes (for example, from the wound exudate) promotes the total formation of pores through these. This would allow skin transpiration and an optimal environment for the wound. 

On the other hand, the mean pore numbers in M4 and CNp‒M4 membranes found in each 0.199 mm^2^ were 4 and 2, respectively. The pore diameter in the M4 membrane was 162.25 ± 40.75 μm, while for CNp‒M4 membranes, this was 73.43 ± 11.04 μm (Figure 5b). Moreover, M4 membranes were significantly thicker and more homogenous in structure than CNp‒M4 membranes. These features could be due to the fact that CNp‒M4 membranes have CNp dispersion in their formulation, which contains Pluronic^®^ F-68, a nonionic surfactant used as a nanoparticle coating that decreases tensile surface in the polymer gel, thus reducing the number and size of the pores formed in the membranes [43].

**Figure 6:**
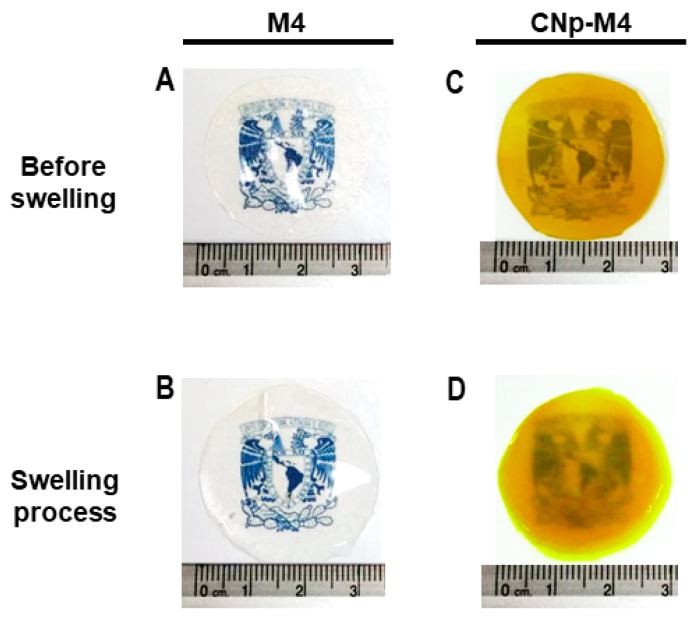
Alginate membranes (M4) before swelling and during the swelling process in PBS medium (**A**,**B**, respectively), and alginate membranes with curcumin nanoparticles (CNp‒M4) before swelling and during the swelling process in PBS medium (**C**,**D**, respectively). Scale in centimeters.

Finally, transparency is an expected feature in our alginate membranes used as wound dressings, in order to observe the possible wound-healing process without removing the dressing. M4 and CNp‒M4 membranes revealed a transparent feature prior to swelling, while CNp‒M4 membranes demonstrated a translucent feature during the swelling process (Figure 6). Although the latter was not completely transparent, it was possible to see through it.

#### 3.2.7. In Vitro Release Study of Drug Dispersion, CNp and CNp‒M4

**Figure 7:**
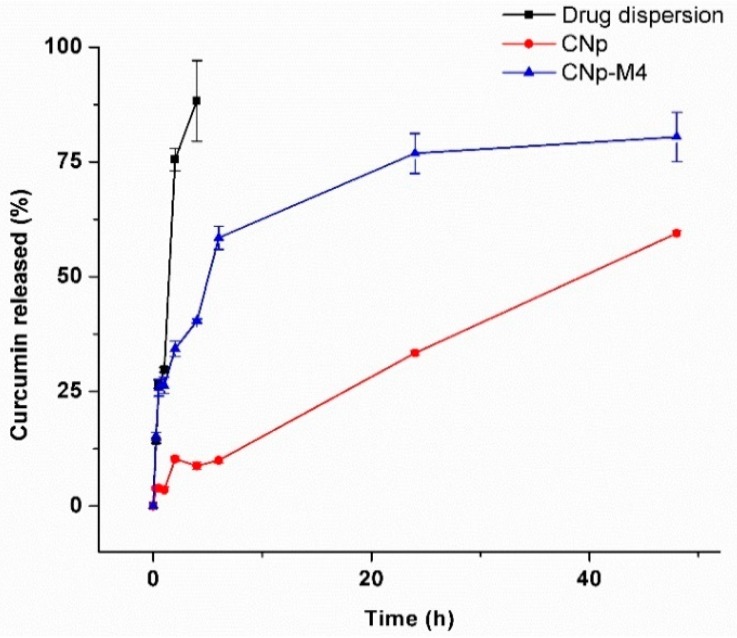
Release profile of curcumin from the drug dispersion, CNp and CNp‒M4 membrane in PBS pH 7.4 (0.1 M, Pluronic^®^ F-127 2% *w*/*v*) at 37 °C. Each point represents the mean ± SE, *n* = 3.

To analyze the mechanism of drug release from the nanoparticles (CNp) and from the nanoparticles inside the membrane (CNp‒M4), an in vitro release study was performed via the dispersion method and is presented in Figure 7. The curcumin release from CNp (red line, circle symbol) showed a low burst effect at 2 h; this behavior could be related to the presence of the curcumin released from the nanoparticles, as well as to the curcumin outside the nanoparticle, inside the border-zone matrix-stabilizer. After that, the release profile exhibited a linear behavior, with nearly 60% of the curcumin released after 48 h of study. Interestingly, in the release profile for CNp‒M4 (blue line, triangle symbol), the burst effect was not evident, and a faster release than CNp (80% of curcumin released at 48 h) was found. These behaviors may be due to the interaction among the membrane excipients, the solvents, and the nanoparticles. To elaborate the CNp‒M4 membrane (Section 2.4.1), the nanoparticles are in contact with water, and the hydrolysis of CNp could be stimulated. In addition, some excipients, such as glycerin and propylene glycol, are co-solvents that could improve the prior solubilization of curcumin. As can be observed, the release from both the CNp and CNp‒M4 membrane was considerably slower than the drug dispersion release (black line, square symbol).

In order to investigate the mechanism of curcumin release from CNp and CNp‒M4, different mathematical models were applied (Table 3). The CNp data were fixed with the Higuchi model (A = 0.0852, B = −0.0459, *R*^2^ = 0.9551), according to those previously reported [44]. This model describes the release of the drug by diffusion from the nanoparticle core into the external solution. On the other hand, the release from CNp‒M4 could be explained with the Korsmeyer–Peppas model, due to the highest squared-correlation-coefficient value being obtained with this method (A = 0.3119, B = −0.5609, *R*^2^ = 0.9536). This model combines the diffusion and erosion mechanisms of the nanoparticles as the explanation for drug release.

### 3.3. Curcumin Permeation Assays

#### 3.3.1. Ex Vivo Permeation 

An ideal system for potential use in chronic diseases with a slow healing process, such as wounds or psoriasis, should exhibit sustained drug release in order to allow permeation through the skin [20,45]. In this regard, the alginate membranes developed in our study possess polymeric networks as a structure, as well as the curcumin encapsulated in PCL nanoparticles dispersed within these networks. These features will allow the slow release of curcumin.

**Figure 8:**
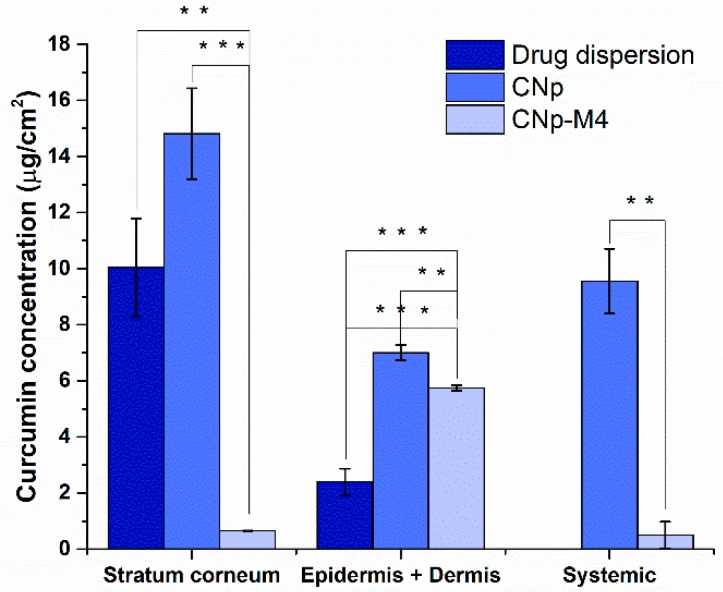
Ex vivo permeation of curcumin after 30 h of treatment with drug dispersion, CNp dispersion, or CNp‒M4 membrane. Stratum corneum-bound particles (obtained from 15 tape strips), epidermis + dermis (surface on which dosed skin was handled after 30 h), and systemic (receptor compartment), mean ± SE, *n* = 4; ** indicates *p* < 0.01 and *** indicates *p* < 0.001 as statistically significant.

An ex vivo permeation study was conducted to determine the distribution of the drug and CNp throughout the stratum corneum, epidermis, and dermis, and to verify whether curcumin can pass through the skin and reach blood circulation after the administration of CNp‒M4 and CNp formulations. With respect to the aqueous dispersion of curcumin (Figure 8), due to the high lipophilic character of the drug, a high accumulation was observed in some superficial layers of the stratum corneum (10.04 ± 1.73 μg/cm^2^). Interestingly, permeation comprises a considerable amount, even from the application of a curcumin dispersion in water, which involves solid particle clusters. This means that the drug particles engage in a dissolution process with the oily components of the stratum corneum, leading to their brief permeation in the superficial region. Although the values are high, there is a higher efficiency of permeation with CNp (14.80 ± 1.61 μg/cm^2^). In the region of the dermis, with a hydrophilic character, the permeated amount of the drug dispersion decreases considerably due to its highly limited solubility in aqueous media (2.40 ± 0.46 μg/cm^2^); this is nearly one third in relation to the CNp value (6.99 ± 0.27 μg/cm^2^). No detection was recorded for the dispersion of the drug that could completely permeate the skin. According to Figure 8, a significant difference was observed between CNp dispersion and CNp‒M4 membrane treatments, with the highest permeation values observed for CNp. The CNp‒M4 membrane treatment revealed the highest amount of curcumin retained in the epidermis and dermis—that is, 5.7 µg/cm^2^ (1.620 ± 0.051% of the total concentration of curcumin)—and the lowest concentration was found in the stratum corneum (SC), at 0.65 µg/cm^2^ (0.140 ± 0.006%). On the other hand, in the CNp dispersion treatment, curcumin was found mostly in the SC; that is, 14.8 µg/cm^2^ (2.62 ± 0.49%) [26], the highest curcumin value in the entire assay.

There was also a significant difference in the amount of curcumin that crossed through the skin (which, in an in vivo model, means reaching the systemic circulation), since the concentration derived from the CNp‒M4 membrane was significantly lower than CNp (0.32% and 2.04% respectively; *p*-value = 0.0019).

The aqueous system of CNp dispersion permitted the curcumin to permeate through the dermis and completely cross the skin. Moreover, CNps have a negative charge; negatively charged nanoparticles permeate the skin more rapidly than positively charged nanoparticles. The skin is predominately negatively charged, and the electrostatic interaction of positive particles with the negatively charged molecules in the skin matrix slows particle diffusion [26]. However, curcumin from CNp‒M4 membranes diffuses more slowly through the skin than that from CNp. These observations may be due to the fact that, in the CNp dispersion, water was used as the medium. Water affects the absorption rates of different substances through the stratum corneum, which is in a constant state of partial hydration under normal conditions. Thus, when immersed in water, dead keratinocytes quickly absorb it, resulting in the pruning effect of the skin [45]. Furthermore, water in contact with skin creates a flow gradient toward the skin’s inner layers, since the inside of the stratum corneum is more hydrated than the surface [46]. On the other hand, the CNp‒M4 membrane does not have a liquid medium in the interface that allows the nanoparticles to flow easily into the deep layers, such as water in the case of the CNp dispersion. Despite this, for the CNp‒M4 membrane, a modulated release is expected because of the degree of swelling of the membrane in response to the presence of exudate. The swelling would permit the relaxation of the polymer chains and the release from the CNp. Otherwise, diffusion from a solid state (CNp‒M4) into a semi-solid state (the skin) would be expected. Therefore, the CNp‒M4 membrane represents a prolonged release system.

In addition, the skin possesses furrows, in which a considerable amount of curcumin is retained, which could not be extracted with the application of adhesive tapes [47]. This curcumin was quantified until mechanical disaggregation. This could explain why the epidermis and dermis had the highest concentration of the drug. 

On the other hand, particle size is an important factor in obtaining the desired therapeutic effect, because nanoparticles with a small size can more easily permeate the physiological barriers; moreover, due to their greater surface, release of the drug is favored [48]. Thus, it should be expected that CNps, with their small size (148.3 nm), and the surfactant effect of Pluronic^®^ F-68 can permeate intercellularly and through hair follicles, favoring accumulation for several hours. In the same way, due to the contact of nanoparticles with the corneocytes of the skin, as well as the prolonged release thereof, a large amount of curcumin was found to be retained in the dermis [24,27]. Although after 30 h the majority of curcumin remained in the stratum corneum when CNp dispersion was applied, the monitoring of the permeation at longer times could allow the observation of a prolonged release system [49]. Therefore, the CNp‒M4 membrane is proposed as a functional prolonged release system for drug delivery in chronic diseases; however, it would be necessary to perform a more prolonged test to observe the diffusion of the drug at a greater proportion.

#### 3.3.2. Permeation Assay in Vivo 

**Figure 9:**
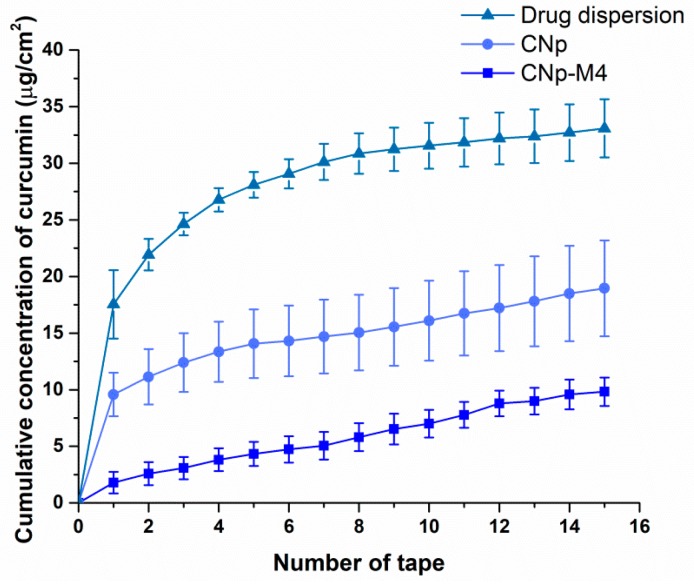
Cumulative concentration of curcumin quantified in the stratum corneum of healthy volunteers. Drug extraction from 15 adhesive tapes applied to the treatment site after placing a CNp‒M4 membrane, CNp dispersion, or drug dispersion for 6 h (mean ± SE; *n* = 4).

In order to evaluate the in vivo skin permeation of curcumin from the CNp‒M4 membrane, CNp dispersion [20], and drug dispersion, a quantification of the curcumin deposited in the stratum corneum by UV–Vis spectrophotometry was performed. The results are presented in Figure 9, according to the work of Goto et al. [50].

Higher permeation values were observed for the drug dispersion in water compared to the CNp and CNp‒M4 membrane, at least in the superficial layers of the stratum corneum, where the tape stripping technique is applied. The higher values of drug permeation could correspond to the high lipophilicity value of the drug, structural symmetry, and low molecular weight. These values coincide with Figure 8: greater drug deposition on the stratum corneum surface, a lower proportion in the dermis due to an inadequate hydrophilic–lipophilic balance, and no recorded quantity that completely permeates the skin. At 6 h after the application of the drug dispersion, CNp‒M4 membrane, and CNp dispersion, the curcumin measured in the stratum corneum reached 33.08 ± 2.57, 9.82 ± 4.23, and 18.96 ± 1.25 μg/cm^2^, respectively [25]. CNp‒M4 remained well adhered during the study, even up to 48 h in other volunteers (data not shown). As can be noted, a greater amount of curcumin was observed in the stratum corneum when the CNp dispersion treatment was applied, compared with the CNp‒M4 membrane. This is because, in the CNp‒M4 membrane, the nanoparticles must be released from the polymeric matrix. This result suggests our formulation as a system of prolonged release that may be useful for long treatments, such as that for a wound (for example, for 7–14 days of application until closure of the lesion). It is noteworthy that the smallest variation observed in permeation values with the CNp‒M4 treatment reveals a system that allows better gradual control release. Moreover, these nanoparticles also possess a stabilizer on the outside, Pluronic^®^ F-68, which interacts through hydrogen bonds with the ‒OH groups of plasticizers and polymers used in the formulation. Thus, when the water makes contact with the membrane, the external part of the polymers swells and promotes the drug—in this case, CNp—to flow outward, permitting its release [51].

With respect to the CNp dispersion, the nanoparticles are free in the medium; thus, they interact more easily with the stratum corneum. Curcumin is encapsulated and uniformly distributed in the PCL nanoparticles, forming nanospheres [52]. The release of curcumin from these latter will depend on the solubility of the drug, the diffusion of curcumin through the matrix of the nanoparticles, thedesorption of curcumin from PCL, the erosion or degradation of the matrix of the nanoparticles, and on the combination of the erosion and diffusion processes [23]. It is also known that, under physiological conditions, a random cleavage of PCL ester bonds occurs, which produces a destabilization of the polymer matrix of the CNp, inducing the release of curcumin [25].

Finally, many studies have reported an accumulation of nanoparticles in hair follicles and the pilosebaceous glands in ex vivo skin experiments [49]. In the same manner, it has been reported that there is better permeation of the drug into the skin, as well as greater absorption in areas with high hair-follicle density. The hair follicle can become a reservoir of substances comparable to the stratum corneum [53]. This means that drugs will penetrate better through the skin of a person with greater amounts of hair follicles. This may explain slight variations in the results obtained, together with variability among individuals (age, body mass, color, and skin moisturization).

## 4. Conclusions

In this study, the development of a new wound dressing for possible application in wound healing was demonstrated. The wound dressing comprises an alginate membrane and PCL nanoparticles stabilized with Pluronic^®^ F-68 with curcumin inside. This new system was designed with a mixture of aqueous plasticizers to confer high-strength mechanical properties. The characterization of the formulation exhibited that it possesses a high absorbency capacity for the removal of possible exudates, transparency for monitoring the wound bed, the presence of pores with controlled dimensions that could facilitate the transpiration of the wound as a synthetic skin substitute, and the gradual release of the drug according to the ex vivo and in vivo studies. In addition, the high adherence of the wound dressing should be noted even in dry skin, as well as its degradation at later times. Therefore, all of these advantages reveal our wound dressing as a good option for wound healing without the need for its removal from the patient.

## Figures and Tables

**Table 1:** Formulation of alginate membranes with different polymers as plasticizers. PVA: poly vinyl alcohol; PVP: polyvinylpyrrolidone; CNp: polycaprolactone (PCL) nanoparticles loaded with curcumin.

Formulation Code	SA (% *w*/*v*)	Polymer		Plasticizer	
		PVA (%*w*/*v*)	PVP (%*w*/*v*)	Gly (%*w*/*v*)	Prop (%*v*/*v*)
	4	2	----	10	----
M2	4	2	----	10	12
M3	4	----	2	10	----
M4	4	----	2	10	12
CNp‒M4	4	----	2	10	12

**Table 2:** Thermal events of curcumin, CNp, and alginate membranes by DSC.

Sample	T_m1_ (°C)	T_m2_ (°C)	T_m3_ (°C)
Curcumin	174	----	----
CNp	63.5	101	212
M4	87	249	----
CNp‒M4	87	233	----

**Table 3:** Squared of the correlation coefficient (R^2^) and coefficients obtained after the linear regression of release data from CNp and CNP‒M4 utilizing four mathematical models.

Mathematical Model	Equation	CNp			CNp‒M4		
		*R* ^2^	A	B	*R* ^2^	A	B
Zero-order	$Q_{t}=Q_{0}+K_{0}t$	0.842	0.5264	4.7385	0.7358	1.2362	31.432
First-order	${\mathrm{lnQ}}_{t}={\mathrm{loQ}}_{0}+K_{1}t$	0.8436	−0.0066	−2.3991	0.8453	−0.0303	−3.1975
Higuchi	$Q_{t}=K_{H}t^{1/2}$	0.9551	0.0852	−0.0459	0.8939	0.1017	0.194
Korsmeyer–Peppas	$\frac{Q_{t}}{Q_{\infty }}={\;K}_{K}t^{n}$	0.9037	0.5422	−1.2655	0.9536	0.3119	−0.5609

Q_t_: amount of drug released in time t; Q_0_: initial amount of drug in the dosage form; Q_∞_: total amount of drug dissolved when the dosage form is exhausted; K_0_, K_1_, K_H_, K_K_: release rate constants; *R*^2^: squared correlation coefficient. y = ax ± b is an equation obtained after regression: a, slope and b, linear coefficient.
